# EGFR and KRAS Mutations in Lung Parenchyma of Subjects With EGFR/KRAS Wild-Type Lung Adenocarcinoma

**DOI:** 10.3389/pore.2021.598292

**Published:** 2021-03-24

**Authors:** Roberto Chalela, Jose Gregorio González-García, Karys Khilzi, Víctor Curull, Albert Sánchez-Font, Raquel Longarón, María Teresa Rodrigo-Calvo, Clara Martín-Ontiyuelo, Joaquim Gea, Beatríz Bellosillo

**Affiliations:** ^1^ Hospital del Mar Medical Research Institute (IMIM), Barcelona, Spain; ^2^ Pompeu Fabra University, Barcelona, Spain; ^3^ Respiratory Medicine Department, Hospital del Mar PSMAR, Barcelona, Spain; ^4^ Centro de Investigación Biomédica en Red Enfermedades Respiratorias (CIBERES), Madrid, Spain; ^5^ Autonomous University of Barcelona, Barcelona, Spain; ^6^ Pathology Department, Hospital del Mar PSMAR, Barcelona, Spain; ^7^ Pathology Department - Molecular Biology Laboratory, Hospital del Mar PSMAR, Barcelona, Spain

**Keywords:** adenocacinoma lung, driver mutation, EGFR–epidermal growth factor receptor, KRAS, Prognosis

## Abstract

The acquisition of driver mutations in non-tumoral cells appears to be very important during the carcinogenesis of adenocarcinoma (ADC). Recent studies suggest that cancer-related mutations may not necessarily be present only in malignant cells, but also in histologically “healthy cells”.

**Objective:** to demonstrate the presence of *EGFR* or *KRAS* mutations in non-tumoral lung cells in subjects with ADC and negative mutational status.

**Results:** mutations in *EGFR* or *KRAS* oncogenes were identified in the normal lung in 9.7% of the subjects. Exon 21 substitution L858R in *EGFR* was detected in two cases while the exon 19 deletion E746-A750 in the *EGFR*, the G12C and G12D substitutions in the *KRAS* were detected once. One patient presented three different mutations in the normal lung parenchyma (*EGFR*_L858R, *KRAS*_G12C and *KRAS*_G12D). The negative-mutation status of the tumor and the mutations detected in the “normal lung” were confirmed using highly sensitive and specific TaqMan PCR (CAST-PCR). No differences were found in terms of progression, progression-free survival or overall survival during the 18 months follow-up.

**Conclusions:** These results confirm the presence of driver mutations in the histologically normal lung parenchyma cells in the absence of mutations coexisting with the primary tumor.

## Introduction

Lung cancer, specifically lung adenocarcinoma (ADC), is frequently diagnosed in an advanced stage with a global 5-years survival not exceeding 17% [1–3]. Even when it is detected at an early stage, the prognosis is poor, especially in terms of tumor recurrence [4, 5]. Pulmonary ADC has an extraordinarily high mutational burden and somatic genomic alterations can be found in more than 75% of the cases. Fortunately, a vast proportion of the oncogenic driver alterations has potential therapeutic implications [6]. The acquisition of driver mutations in still histologically non-tumoral cells appears to be very important during the ADC carcinogenesis since it will potentially lead to a clonal cell expansion [7]. Moreover, two studies have demonstrated the presence of cancer-related mutations in non-tumoral cells of subjects with endometriosis and arteriovenous malformations of the brain [8, 9]. These findings demonstrate that cancer-related mutations may not necessarily be present only in malignant cells, but also in histologically non-tumoral cells. Our group has recently demonstrated that subjects with localized lung ADC with epidermal growth factor receptor-*EGFR* or *KRAS* proto-oncogene alterations showed the same driver mutation in the non-tumoral lung tissue as far as a 21.3% of the cases [9]. These findings were associated with a significantly lower disease-free survival at 12 months and are in line with other previously published evidence related with molecular alterations in non-tumoral lung [10]. Our hypothesis is that cancer-related mutations can appear in histologically non-cancerous cells even in the absence of molecular alterations in the primary tumor during the field cancerization process. Accordingly, the aim of the present study was to demonstrate the presence of *EGFR* or *KRAS* mutations in non-tumoral lung cells even in subjects with early-stage ADC with negative mutational testing.

## Methods

### Subjects

Subjects with early-stage lung ADC with negative mutational status and candidates for curative surgical resection were prospectively recruited in our center, a tertiary teaching hospital. Tumor and histologically normal lung parenchyma samples were obtained and processed. Thirty-five subjects with *EGFR* mutation-negative and *KRAS* mutation-negative lung adenocarcinoma were included. The normal lung parenchyma (NLP) sample was defined as a histologically normal tissue with complete absence of micro-tumor invasion assessed by a pathologist and obtained in the area of the resected lung furthest from the tumor. Finally, viable non-tumoral DNA was obtained in 31 of these subjects and a competitive allele-specific TaqMan PCR was performed to identify the presence of *EGFR* or *KRAS* mutations. The cohort was followed-up during 18 months and clinical data were collected for months 1, 2, 6, 12 and 18. The study was designed and carried out in accordance with the ethical guidelines of the Declaration of Helsinki and European legislation, and the procedure was approved by our Ethics Committee. Informed consent was obtained from all individuals.

### Tumor DNA Extraction and Sequencing

The molecular study of the tumor was carried out after surgery and included the detection of EGFR using TheraScreen PCR and direct sequencing for KRAS alterations. The commercially kit QIAamp DNA Mini kit (Qiagen, Hilden, Germany) was used for DNA extraction. The *EGFR* mutations were detected using the commercial real-time PCR TheraScreen *EGFR* RGQ PCR kit (Qiagen). This is a highly sensitive assay based on Scorpions^®^ real-time PCR technology and mutation specific ARMS^®^ primers that detect 29 different somatic mutations in the gene. In addition, exon 2 of the *KRAS* gene and exons 18, 19, 20, 21 of the *EGFR* gene were studied with Sanger sequencing, using BigDye v3.1 (Applied Biosystems, Foster City, CA, United States), being assessed on the 3500DX Genetic Analyzer (Applied Biosystems).

### Normal Lung Parenchyma DNA Extraction and Sequencing

DNA was extracted from two sections of normal appearing lung samples using the QIAamp DNA Mini kit (Qiagen). Competitive allele-specific TaqMan PCR (CAST-PCR, Applied Biosystems, 4465804) was performed in order to determine *KRAS* and *EGFR* mutational status. Samples were amplified in duplicate in independent experiments with TaqMan™ Genotyping Master Mix (ThermoFisher) using the following assays: *KRAS* p.G12C—Hs00000113_mu; *KRAS* p.G12V—Hs00000119_mu; *KRAS* p.G12D—Hs00000121_mu; *KRAS* p.G12A—Hs00000123_mu; *KRAS* p.G12R—Hs00000117_mu; and *KRAS* p.G13C—Hs00000125_mu, *EGFR* exon 19 deletions—Hs00000228_mu; *EGFR* p.L858R—Hs00000102_mu; *EGFR* p.T790M—Hs00000106_mu and G719A—Hs00000104_mu. PCR amplifications were performed in a 7500Fast real-time PCR system (Applied Biosystems). All experiments have been confirmed and the technique complies with the Minimum Information for Publication of Quantitative Real-time PCR Experiments (MIQE).

### Statistical Analysis

Categorical variables were described as frequencies and percentages, whereas continuous variables as mean ± standard deviation. Pearson’s Chi-Square or Fisher exact tests were used to compare categorical variables. The non-parametric Mann-Whitney *U* test was used to assess differences between groups. *p* values ≤0.05 were considered statistically significant. Analyses were performed with SPSS 21.0.

## Results

The main clinical, functional and tumor characteristics of the cohort are shown in Table 1. All subjects were stratified in stages based on the present TNM classification (IASLC, 8th edition) and only stage I or II subjects were included [3]. The surgical procedures were performed in accordance with the institution clinical-practice recommendations. The most common procedure was a lobectomy (67.7%) followed by segmentectomy (22.6% and bilobectomy (9.7%). Almost all the subjects (30 of 31, 96.8%) were smokers or former smokers.

**TABLE 1:** Baseline characteristics and comparison between mutated NLP and non-mutated NLP.

	Total n = 31	Mutated NLP n = 3	Non-mutated NLP n = 28	*p* value
Age, mean (SD), yrs	64.2 (7.2)	60 (6)	64.5 (7.1)	0.29
Current or former smoker, n (%)	30 (96.8)	2 (66.7)	28 (100)	0.00
Smoking index, mean (SD), pack-year	53.2 (23)	40 (34.6)	54.6 (22)	0.30
*Sex, n* (*%*)				
Male	25 (81)	1 (33)	24 (86)	0.02
Female	6 (19)	2 (67)	4 (14)	
*Comorbidities, n* (*%*)				
Previous cancer	12 (38.7)	1 (33.3)	11 (39.3)	0.84
Dyslipidemia	10 (32.3)	1 (33.3)	9 (32.1)	0.96
Hypertension	10 (32.3)	0 (0)	10 (35.7)	0.20
Diabetes mellitus	7 (22.6)	1 (33.3)	6 (21.4)	0.63
Alcoholism	9 (29)	0 (0)	9 (32.1)	0.24
COPD	8 (25.8)	0 (0)	8 (28.6)	0.28
Ischemic cardiomyopathy	2 (6.5)	0 (0)	2 (7.1	0.63
Chronic kidney disease	1 (3.2)	0 (00)	1 (3.6)	0.73
*Lung function tests, mean* (*SD*)				
FEV_1_ *, % ref*	74.5 (16.1)	81 (31.1)	74.2 (15.3)	0.56
FVC*, % ref*	86.9 (16.6)	85.5 (28.9)	86.5 (16.1)	0.93
TLC*, % ref*	99.9 (13)	77	99.8 (13)	0.09
RV/TLC*, %*	46.2 (11)	38	46.2 (11)	0.47
DLCO*, % ref*	65.3 (18.8)	79.5 (47)	64.4 (16.4)	0.27
*Karnofsky scale, mean* (*SD*)	93.3 (6)	100	92.8 (6)	0.09
*Tumor characteristics*				
SUV By PET, mean (SD), cm	6.5 (4.7)	3.4 (2.5)	6.8 (4.7)	0.33
T (tumor size), mean (SD), cm	2.8 (18.4)	1.4 (0.1)	2.9 (1.8)	0.15
N (nodal infiltration), n (%)	3 (9.7)	1 (33.3)	2 (7.1)	0.14
M (metastasis), n (%)	0 (0)	0 (0)	0 (0)	—
*Post-operative stage groups, n* (*%*)				
I	23 (74.2)	21 (75)	2 (66.7)	0.75
II	8 (25.8)	1 (33.3)	7 (25)	0.75
III - IV	0 (0)	0 (0)	0 (0)	—
*Diagnostic tests, n* (*%*)				
PET-CT scan	27 (87.1)	2 (66.7)	25 (89.2)	0.77
Endobronchial ultrasound (EBUS)	17 (54.8)	2 (66.7)	15 (53.6)	0.76

Abbreviations: NLP, normal lung parenchyma; SD, standard deviation; COPD, chronic obstructive pulmonary disease; FEV_1_, forced expiratory volume in the first second; FVC, forced vital capacity; TLC, total lung capacity; RV, residual volume; DLco, transfer coefficient for CO; SUV, standardized uptake value; PET, positron emission tomography.

We identified five mutations in *EGFR* or *KRAS* oncogenes in the normal lung parenchyma among three subjects (9.7%). The exon 21 substitution L858R in *EGFR* was detected in two cases while the exon 19 deletion E746-A750 in the *EGFR*, the codon 12 substitution G12C and G12D in the *KRAS* were detected once. Surprisingly, in one patient, three different mutations were identified in NLP (*EGFR*_L858R, *KRAS*_G12C and *KRAS*_G12D). More details of the three subjects with mutated NLP can be found in Table 2.

**TABLE 2:** Detailed mutation characteristics and progression.

N°	Age (years)	Sex	TNM	Mutational status in NLP	Distant - local progression	Site of progression
1	69	M	T2AN0M0	*KRAS* Wild-type	NO	
				*EGFR* Wild-type		
2	67	M	T3N1M0	*KRAS* Wild-type	NO	
				*EGFR* Wild-type		
3	56	M	T2AN0M0	*KRAS* Wild-type	YES	Adrenal
				*EGFR* Wild-type		
4	55	M	T1BN0M0	*KRAS* Wild-type	NO	
				*EGFR* Wild-type		
5	62	M	T2AN1M0	*KRAS* Wild-type	YES	Lymph Nodes
				*EGFR* Wild-type		Local progression
6	58	M	T1BN0M0	*KRAS* Wild-type	NO	
				*EGFR* Wild-type		
7	59	M	T1AN0M0	*KRAS* Wild-type	YES	Brain
				*EGFR* Wild-type		Adrenal
8	67	M	T3N0M0	*KRAS* Wild-type	NO	
				*EGFR* Wild-type		
9	55	M	T1AN0M0	*KRAS* Wild-type	NO	
				*EGFR* Wild-type		
10	67	M	T1BN0M0	*KRAS* Wild-type	NO	
				*EGFR* Wild-type		
11	55	M	T3N0M0	*KRAS* Wild-type	YES	Brain
				*EGFR* Wild-type		Local progression
12	69	M	T2AN0M0	*KRAS* Wild-type	NO	
				*EGFR* Wild-type		
13	78	M	T2AN0M0	*KRAS* Wild-type	NO	
				*EGFR* Wild-type		
14	75	M	T1BN0M0	*KRAS* Wild-type	YES	Lymph Nodes
				*EGFR* Wild-type		Bones
15	73	M	T3N0M0	*KRAS* Wild-type	NO	
				*EGFR* Wild-type		
16	66	M	T1BN0M0	*KRAS* Wild-type	NO	
				*EGFR* Wild-type		
17	53	F	T1AN0M0	*KRAS* Wild-type	NO	
				*EGFR* Wild-type		
18	60	M	T1AN0M0	*KRAS* Wild-type	NO	
				*EGFR* Wild-type		
19	60	F	T2AN0M0	*KRAS* Wild-type	NO	
				*EGFR* Wild-type		
20	59	M	T1AN0M0	*KRAS* Wild-type	NO	
				*EGFR* Wild-type		
21	53	M	T1AN0M0	*EGFR* deletion E746-A750	NO	
				*KRAS* Wild-type		
22	64	F	T1AN0M0	*KRAS* Gly12Cys	YES	Liver
				*KRAS* Gly12Asp		
				*EGFR* substitution L858R		
23	63	F	T1BN0M0	*KRAS* Wild-type	NO	
				*EGFR* Wild-type		
24	72	M	T1AN0M0	*KRAS* Wild-type	NO	
				*EGFR* Wild-type		
25	68	M	T1AN0M0	*KRAS* Wild-type	NO	
				*EGFR* Wild-type		
26	61	F	T1BN0M0	*KRAS* Wild-type	NO	
				*EGFR* Wild-type		
27	70	M	T1AN0M0	*KRAS* Wild-type	NO	
				*EGFR* Wild-type		
28	67	M	T1AN0M0	*KRAS* Wild-type	YES	Lymph Nodes
				*EGFR* Wild-type		
29	80	M	T2AN0M0	*KRAS* Wild-type	YES	Adrenal
				*EGFR* Wild-type		
30	65	M	T2AN0M0	*KRAS* Wild-type	YES	Brain
				*EGFR* Wild-type		
31	63	F	T1AN0M0	*EGFR* substitution L858R	NO	
				*KRAS* Wild-type		

Abbreviations: *EGFR*, Epidermal Growth Factor Receptor gene; *KRAS*, Kirsten Rat Sarcoma viral oncogene homolog.

**FIGURE 1:**
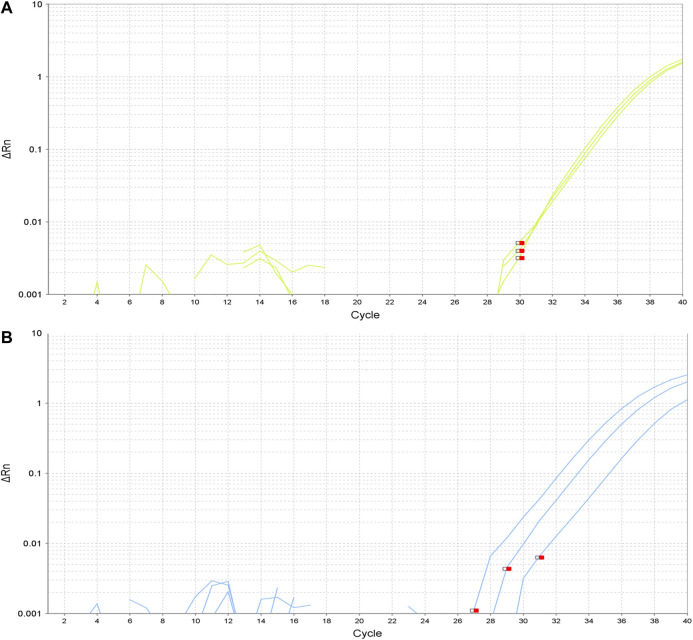
Amplification plots of detected mutations. **(A)**. Amplification plot for EGFR exon 19 deletion E746-A750. **(B)**. Amplification plot for EGFR exon 21 substitution L858R.

In these three subjects, the negative-mutation status of the tumor for the specific mutation detected in the NLP was confirmed using highly sensitive and specific TaqMan PCR (CAST-PCR). The confirmation of the positive-mutation status in the NLP was also performed for the previously mentioned five specific mutations. In all the assays, the PCR efficiency was between 95 and 105%. To improve specificity and avoid false positives we only considered as positives the assays for *EGFR* mutations when the amplification occurred before the cycle 35. For the two *KRAS* mutations detected, the amplification occurred between the cycle 35 and 38. The mutation was confirmed in both cases. The amplification plots are shown in Figure 1. Real-time PCR results for positive cases were confirmed in independent experiments. We have also performed digital PCR (dPCR) to confirm the presence of the mutations reported and, in addition, with the samples with enough material, we have repeated the qPCR assays.

### Mutated NLP vs. Non-mutated NLP: Clinical Outcomes, Recurrence and Survival

We only found differences between both groups in terms of tobacco status and gender, but considering the limited number of cases, we cannot draw any conclusion. Data from both groups are shown in Table 1.

One patient died in the post-operative setting. During the 18 months follow-up, two subjects died within the non-mutated NLP group, while none died in the other group. No differences were found in terms of progression (locally or at distance), progression-free survival or overall survival between both groups during the follow-up.

## Discussion

This study confirms the presence of driver mutations in the histologically normal lung parenchyma cells coexisting with the absence of mutations in the primary tumor. Our findings are consistent with the hypothesis that during the carcinogenesis process multiple cells can gain somatic mutations without necessarily producing a clonal expansion. In a previous study, we confirmed that the same driver mutation detected in the lung adenocarcinoma was also present in non-tumoral samples in a fifth of the subjects. However, this study has the limitation that this detection could be secondary to contamination by tumor DNA from blood or tumor cells, which may not have been detected by the usual histopathological methods. Although this limitation was unlikely, it could not be one hundred percent ruled out. In the present this study, having excluded the presence of mutations in *EGFR* or *KRAS* by highly specific and sensitive techniques in the primary tumor, we are able to confirm that the mutations detected in other lung cells were not the result of contamination from the tumor. These findings make us change the way we understand and define a driver-mutation.

The prevalence of *EGFR* and *KRAS* mutations detected in the present study is only 9.7%, lower than the one detected in our previous study (21.3%) and in those studies on endometriosis (26%) and arteriovenous malformations of the brain (48%). The prevalence observed in the present study is likely to be higher if more extensive molecular studies that included other molecular alterations were carried out. Additionally, to stress our ambitious hypothesis, we decided to increase the specificity not including the mutations occurred in later cycles of PCR as well as to exclude the substitution of T790M in exon 20 that are usually considered as secondary mutations in the final analysis.

In the present study, we could not find differences in clinical outcomes such as recurrence or disease-free survival, between the two groups, possibly because of the sample size. One patient (33.3%) in the group of the mutated NLP presented progression at 18 months in the form of hepatic metastases; while in the non-mutated NLP group 8 (28.6%) did, not reaching however statistical significance. Subjects in the mutated NLP group were significantly less-frequent smokers and predominantly women when compared with the non-mutated NLP group, this is in line with the evidence that is consistent with the higher prevalence of driver-mutations in non-smoker women.

Interestingly, one patient presented three different mutations in the NLP sample wich included one in the *EGFR* and two in the *KRAS*. Normally the mutations in *EGFR* and *KRAS* are considered as mutually exclusive mutations in lung cancer. Although this finding is surprising, it does not seem improbable either, since, unlike a tumor, where all the cells come from the clonal expansion of a single malignant cell, the normal lung parenchyma samples may contain multiple different cell lines. Our results support our prior hypothesis that molecular changes can occur in multiple cells even without malignancy changes.

## Limitations

There are multiple limitations in the present study, mainly the small sample size, although this limitation does not affect the main objective of the study, which was to demonstrate the presence of driver-mutations in the absence of tumor mutations. The sample size surely affects clinical outcomes, which was not the objective of the study. Contamination or sample mix-up of the studied samples with tumor cells that contain molecular alterations has always been a topic of discussion, but we consider it highly unlikely for several reasons. First, by ruling out molecular alterations in the tumor, contamination would be much less relevant. Second, a “non-tumoral lung” sample was considered when resection margins were tumor-free assessed by an expert pathologist and the area of lung farthest from the tumor was selected for the molecular analysis.

## Data Availability

The raw data supporting the conclusions of this article will be made available by the authors, without undue reservation.
